# Population Studies and Carrageenan Properties in Eight Gigartinales (Rhodophyta) from Western Coast of Portugal

**DOI:** 10.1155/2013/939830

**Published:** 2013-10-27

**Authors:** Leonel Pereira

**Affiliations:** Institute of Marine Research (IMAR-CMA), Department of Life Sciences, Faculty of Sciences and Technology, University of Coimbra, 3001-455 Coimbra, Portugal

## Abstract

Eight carrageenophytes, representing seven genera and three families of Gigartinales (Florideophyceae), were studied for 15 months. The reproductive status, dry weight, and carrageenan content have been followed by a monthly random sampling. The highest carrageenan yields were found in *Chondracanthus acicularis* (61.1%), *Gigartina pistillata* (59.7%), and *Chondracanthus teedei* var. *lusitanicus* (58.0%). Species of Cystocloniaceae family produces predominantly iota-carrageenans; Gigartinaceae family produces hybrid kappa-iota carrageenans (gametophytic plants) and lambda-family carrageenans (sporophytic plants); Phyllophoraceae family produces kappa-iota-hybrid carrageenans. Quadrate destructive sampling method was used to determine the biomass and line transect. Quadrate nondestructive sampling method, applied along a perpendicular transect to the shoreline, was used to calculate the carrageenophytes cover in two periods: autumn/winter and spring/summer. The highest cover and biomass were found in *Chondrus crispus* (3.75%–570 g/m^2^), *Chondracanthus acicularis* (3.45%–99 g/m^2^), *Chondracanthus teedei* var. *lusitanicus* (2.45%–207.5 g/m^2^), and *Mastocarpus stellatus* (2.02%–520 g/m^2^).

## 1. Introduction

 Carrageenans are industrially important hydrocolloids that are found in various red seaweeds (Gigartinales, Rhodophyta) [1, 2]. Carrageenans are a family of water soluble, linear, and sulfated galactans. They are composed of alternating 3-linked *β*-d-galactopyranose (G-units) and 4-linked *α*-d-galactopyranose (D-units) or 4-linked 3,6-anhydro-*α*-d-galactopyranose (DA-units), forming the disaccharide repeating unit of carrageenans. The most common types of carrageenans are traditionally identified by a Greek prefix and more recently by the letter codes developed by Knutsen and collaborators [3]. The three commercially most important carrageenans are called iota-, kappa-, and lambda-carrageenan. The letter codes of these carrageenan types are G4S-DA, G4S-DA2S, and G2S-D2S,6S, respectively. Kappa-carrageenan is present, over all, in the species pertaining to the *Hypneae* (Hypnaceae) and *Kappaphycus* genera (Solieriaceae) and the species belonging to the *Eucheuma* genus (Solieriaceae), are the principal source of iota-carrageenan [4–6]. Kappa/iota-hybrid carrageenans are found in the gametophytic life phases of several species in the families of Gigartinaceae and Phyllophoraceae [7–9]. The mu- and nu-carrageenan, existing in the native phycocolloid samples, are the biologic precursors of kappa- and iota-carrageenan [10]. *In vivo*, iota- and kappa-carrageenan are formed enzymatically from the precursor carrageenans by a sulfohydrolase [11, 12]. *In vitro*, these precursor residues are converted to the corresponding gelling carrageenan on treatment with alkali. Alkali extraction is commonly used in the commercial production of kappa- and iota-carrageenan to increase the 3,6-anhydro-D-galactose content, since this results in a product with enhanced gelling properties [13, 14]. The tetrasporic life phase of Gigartinaceae contains carrageenans of the lambda family. In general, carrageenan serves as a gelling (kappa-family carrageenans), stabilizing, and viscosity-building agent (lambda-family carrageenans) in food products, pharmaceutical formulations, cosmetics, and oil well drilling fluid [2, 15].

The species *Chondrus crispus* and *Mastocarpus stellatus* were the first seaweed used for carrageenan extraction. Due to the fact that both species live in the same biotype, their harvest is generally simultaneous. Although *C. crispus* may occur in considerable quantities to a depth of 12 m in the sublittoral zone of the maritime provinces of Canada, in Europe, it is found mainly in intertidal rock pools [16, 17]. *M. stellatus* is also found in rock pools, mostly in the intertidal zone [16]. In the Northeast Atlantic, both species are found from North Cape (Norway) to Mauritania [17].

Generally, the harvest is made during a period depending on the local customs, climate, and sea behaviour. In Portugal, *C. crispus* and *M. stellatus* have been exploited, especially in the north (Minho, Douro, and Beira Litoral) [18–20]. The carrageenophytes are plucked from the intertidal zone during summer, sun dried, sold to concentrators, and then exported [19, 20]. The collection is controlled by regulations, which first date from 1909, that specify periods of seaweeds harvesting from July to November and require the issuing of licences for each area of the coastline and control prices [16, 19].

Population studies and carrageenan content have recently been performed on wild populations of *Chondrus crispus* and *Mastocarpus stellatus*, the main species with industrial use and harvested in Galicia (Spain) [21, 22].

In 1955, all the carrageenans were gotten from *C. crispus* and *M. stellatus*. Today, these species represent no more than 10% of the total harvest. Most of the currently used seaweeds in the world are cultivated species belonging to the genera *Eucheuma* and *Kappaphycus* as sources of iota- and kappa-carrageenan, respectively [2, 9].

Large carrageenan processors have fuelled the development of *Kappaphycus alvarezii *(which goes by the name “cottonii” to the trade) and *Eucheuma denticulatum *(commonly referred to as “spinosum” in the trade), farming in several countries including the Philippines, Indonesia, Malaysia, Tanzania, Kiribati, Fiji, Kenya, and Madagascar [23]. Indonesia has recently overtaken the Philippines as the world 's largest producer of dried carrageenophyte biomass [15]. 

Shortages of carrageenan-producing seaweeds suddenly appeared in mid-2007, resulting in doubling of the price of carrageenan; some of this price increase was due to increased fuel costs and a weak US dollar (most seaweed polysaccharides are traded in US dollars). The reasons for shortages of the raw materials for processing are less certain; perhaps it is a combination of environmental factors. The drop in production could be also due to a depletion of natural resource caused by a degradation of the habitat and the overexploitation. Most hydrocolloids are experiencing severe price movements. The average prices of carrageenans were US$ 10.5/ kg^−1^, and the global sales in 2009 were US$ 527 million [9, 15, 24].

The present study was carried out in order to evaluate the population and phycocolloid ecology of several underutilized Gigartinales: *Chondracanthus teedei* var. *lusitanicus* (Rodrigues) Bárbara *et* Cremades (Gigartinaceae), *Chondracanthus acicularis *(Roth) Fredericq (Gigartinaceae), *Gigartina pistillata* (S.G. Gmelin) Stackhouse (Gigartinaceae), *Calliblepharis jubata* (Goodenough *et* Woodward) Kützing (Cystocloniaceae), *Gymnogongrus crenulatus* (Turner) J. Agardh (Phyllophoraceae), and *Ahnfeltiopsis devoniensis* (Greville) P.C. Silva *et* DeCew (Phyllophoraceae) and to compare them with the traditionally harvested carrageenophytes *Chondrus crispus* Stackhouse (Gigartinaceae) and *Mastocarpus stellatus* (Stackhouse) Guiry (Phyllophoraceae). To achieve this goal, a natural population of mixed carrageenophytes situated at Buarcos bay (central north of the Portuguese Atlantic coast) was studied during 15 months. To determine the nature of the produced phycocolloid, we examined and quantified the native and alkali-modified carrageenan extracted from the different phases of life history of the studied carrageenophytes (tetrasporophyte, female gametophyte, and nonfructified thalli) with FTIR-ATR, FT-Raman, and ^1^H-NMR. The nature of the polysaccharides (without any type of extraction) present in these seaweeds was determined with FTIR-ATR and FT-Raman analysis of the dry ground seaweed [8, 25, 26].

## 2. Material and Methods

A representative population of the eight studied carrageenophytes, localised at Buarcos bay (40°10′5.99′′N, 8°53′22.27′′W) in the Northern Portuguese coast, was investigated for about 15 months. The plants were collected from a rocky-shore substrate, with numerous sand basins, in the intertidal zone. At each sampling time, pH, salinity, surface water, and air temperature were recorded.

Carrageenophytes coverage was estimated in two periods (autumn/winter and spring/summer), using a modification of the “Braun-Blanquet” scale [27, 28]. A 100 cm (1 m^2^) quadrate, applied along a perpendicular transect (100 m) to the shoreline, was used to evaluate the carrageenophytes cover. 

For determination of biomass and thalli length, eight quadrates (10 × 10 cm) were randomly positioned in the extensive beds of carrageenophytes and destructively sampled [29–31]. The samples were rinsed in distilled water and dried in ventilated oven to constant weight (60°C). Biomass was expressed as a dry weight per square meter of substrate.

The percentage of each lifecycle phase, dry weight, and carrageenan content was evaluated. For these determinations, 100 individuals, larger than 3 cm, of each species were collected at random, monthly. At the laboratory, carrageenophytes fronds were sorted into the different lifecycle phases and then rinsed in distilled water to eliminate debris and salt on the thalli surfaces and dried, in a ventilated oven, to constant weight at 60°C. Carrageenan extraction was carried out according to the process described by Pereira and collaborators [25, 32].

Data on plant size, biomass, lifecycle phase, dry weight, and yields were presented as average ± standard error (with *n* = number of samples used in the study). One-way ANOVA (considering three values of *P*: significant, *P* < 0.05, very significant, *P* < 0.01, and highly significant, *P* < 0.001) of plant size, biomass, lifecycle phase, dry weight, and carrageenan yields was made to analyzse possible variances between seasons [33].

Samples of ground, dried algal material were analysed by FTIR-ATR and FT-Raman [8, 25, 26, 32] for the determination of native phycocolloid composition. The FTIR-ATR spectra of ground, dried seaweed, native and alkali-modified carrageenan were recorded on an IFS 55 spectrometer, using a Golden Gate single-reflection diamond ATR system, with no need for sample preparation. All spectra are the average of two counts, with 128 scans each and a resolution of 2 cm^−1^. The room-temperature FT-Raman spectra were recorded on a RFS-100 Bruker FT-spectrometer, using a Nd:YAG laser with excitation wavelength of 1064 nm. Each spectrum is the averaging of two repeated measurements of 150 scans each and a resolution of 2 cm^−1^.

^1^H-NMR spectra were taken on a Bruker AMX600 spectrometer operating at 500.13 MHz at 65°C. Typically, 64 scans were taken with an interpulse delay of 5 s (*T*_1_ values for the resonance of the anomeric protons of kappa- and iota-carrageenan are shorter than 1.5 s). Sample preparation for the ^1^H-NMR experiments involved dissolving the carrageenan sample (5 mg mL^−1^) at 80°C in D_2_O, containing 1 mM TSP (3-(trimethylsilyl) propionic-*2,2,3,3*-*d*_4_ acid sodium salt) and 20 mM Na_2_HPO_4_, followed by sonication for 1 h three times in a sonicator bath (Branson 2510). Chemical shifts (*δ*) are referred to the internal TSP standard (*δ* = −0.017 ppm) relative to the IUPAC recommended standard DSS for ^1^H according to van de Velde and collaborators [34] and Pereira and van de Velde [1]. Assignments of the ^1^H-NMR spectra were based on the chemical shift data summarised by van de Velde and collaborators [14, 34].

## 3. Results and Discussion

**Figure 1:**
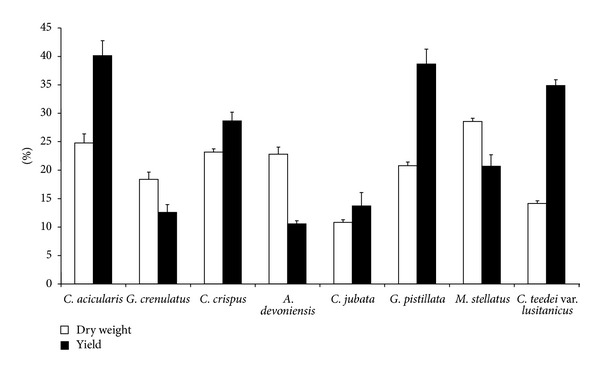
Carrageenophytes dry weight expressed as the percentage of fresh weight and carrageenan yields expressed as the percentage of dry weight (average ± standard error, *n* = 13).

**Figure 2:**
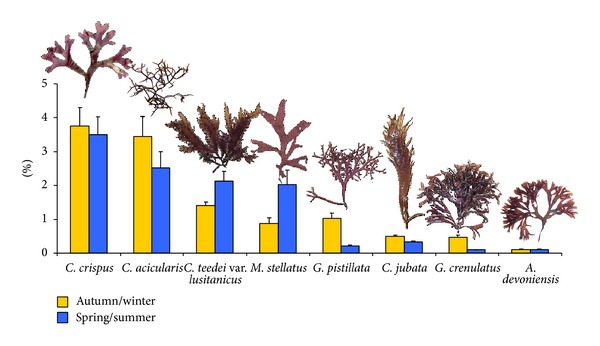
Carrageenophytes coverage in autumn/winter and spring/summer (average ± standard error, *n* = 46).

Table 1 shows the most significant phycocolloid parameters: harvest season, lifecycle phase, yields, and carrageenan composition. Carrageenophytes cover, dry weight, and carrageenan content are presented in Figures 1 and 2, respectively.

### 3.1. Physical-Chemical Data

In Buarcos bay, the average water temperature ranged from 12°C in autumn/winter to 22°C in spring/summer, and the mean air temperature varied from 10°C to 23°C between these periods. In contrast, the pH and salinity have not changed significantly between seasons, with average values of 8.3 and 32.8 S‰, respectively.

### 3.2. Cover Biomass, and Plant Size

*C. crispus* is the dominant species regarding the coverage (Figure 2) and the available biomass for harvesting. The highest values of biomass (570 g/m2) and carrageenan content (see Table 1) have been registered in spring/summer. The maximum average length was 13.8 ± 1.2 cm (*n* = 100) in summer and a minimum of 8.2 ± 0.5 cm (*n* = 100) in winter.

Although it is only the fourth seaweed in terms of cover (Figure 2), *M. stellatus* shows a high biomass (520 ± 2.0 g/m^2^, *n* = 8) in spring/summer. The average length of this species was 6.3 ± 0.5 cm (*n* = 13), with a maximum of 9.5 ± 1.2 cm (*n* = 100) in summer and a minimum of 4.1 ± 0.8 cm (*n* = 100) in winter. The data on seasonal variation length show statistical significance (one-way ANOVA, *P* < 0.001).

In spite of not being a harvested seaweed, it is surprising that, among the carrageenophytes studied, *C. teedei* var. *lusitanicus* is one of those which presents the highest values of average carrageenan yields (Figure 1), cover (Figure 2), and biomass (594 ± 10.5 g/m^2^) in spring/summer. The maximum average length was 7.7 ± 0.4 cm (*n* = 100) in summer and a minimum of 2.9 ± 0.2 cm (*n* = 100) in winter. The biomass values ranged between 110 ± 1.9 g/m^2^ (*n* = 8) in summer and 594 ± 10.5 g/m^2^ (*n* = 8) in late spring. Biomass and length showed low values in autumn and winter, a small increase occurred in early spring, and the highest values of these parameters were recorded in early summer.

The highest carrageenan yields, in this study, were recorded in *G. pistillata,* with an average value of the 38.7% (Figure 1). Nevertheless, the coverage and biomass are relatively low (Figure 2). The second highest coverage was found in *C. acicularis *(Figure 2), but this carrageenophyte presents a low biomass mainly in spring/summer (22 ± 0.2 g/m^2^, *n* = 8). This carrageenophyte presents an isomorphic triphasic lifecycle, but the nonfertile thalli and the tetrasporophytes are difficult to distinguish from one another, and consequently these reproductive phases were not separated. Due to the thalli nature, very thin and tangles, the data on plant size were not done. The cover (Figure 2) and biomass are very low in the remaining carrageenophytes: *C. jubata*, *G. crenulatus,* and *A. devoniensis*. 

### 3.3. Lifecycle Phase

**Figure 3:**
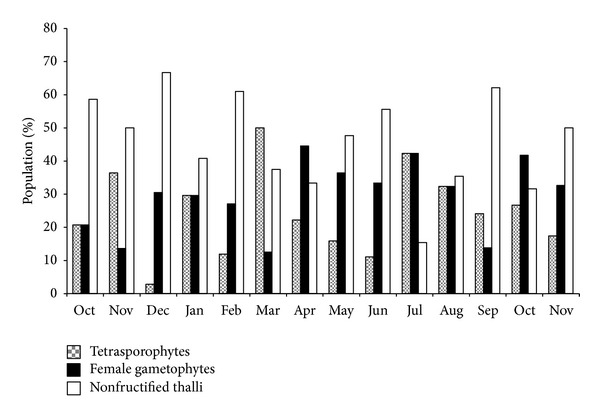
Population structure of C*hondrus crispus *in Buarcos bay.

*C. crispus* shows an isomorphic triphasic lifecycle [35–37], although in the studied population, the nonfructified thalli were dominant in most samples (see Figure 3), and the percentage varied from 15.4% (summer) to 66.7% (winter). The female gametophytes were present in all samples, varying the proportion from 12.5% (late winter) to 44.5% (spring). The tetrasporophytes were also present in all samples, with a maximum of 50% in spring and a minimum of 11.1% in summer. Compared with female gametophytes (cystocarpic thalli) (29.4 ± 2.9%, *n* = 14), the tetrasporophytes (tetrasporic thalli) are usually less abundant (24.5 ± 3.4%, *n* = 14); the predominance of gametophytes in *C. crispus* populations was also found in other works [37–39]. The average percentage of nonfructified thalli was 46.1 ± 3.9% (*n* = 14). The data on seasonal variation in the percentage of individuals of each generation have statistical significance (one-way ANOVA, *P* < 0.001).

*G. pistillata* is the type of species of the *Gigartina* genus [40, 41]; despite possessing an isomorphic triphasic lifecycle [42], it shows heterosporic thalli (i.e., producing both tetraspores and carpospores) [43, 44], which were found in all studied samples. The tetrasporophytes were dominant in most samples, and the percentage varied from 10% (spring) to 81.8% (late autumn). The female gametophytes were present in all samples, varying the proportion of 6.8% (late autumn) to 90% (spring). The heterosporic thalli were present in all samples, with a maximum of 30.8% in early winter and a minimum of 1.9% in summer. The data on seasonal variation in the percentage of individuals of each generation have statistical significance (one-way ANOVA, *P* < 0.001).

*C. acicularis* presents an isomorphic triphasic lifecycle [41], but the nonfructified thalli and the tetrasporophytes are difficult to distinguish from one another, and consequently these reproductive phases were not separated.

**Figure 4:**
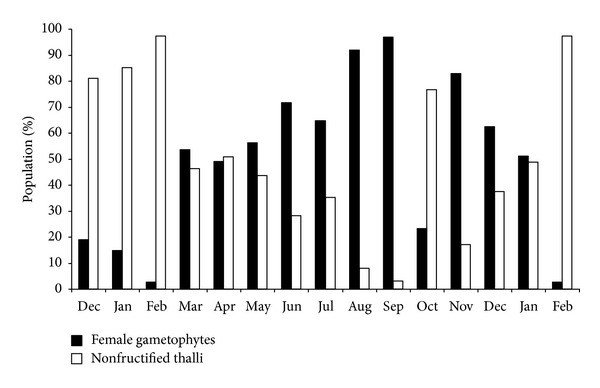
Population structure of *Mastocarpus stellatus* in Buarcos bay.

*M. stellatus* presents a heteromorphic triphasic lifecycle with a sporophytic crust, formerly *Petrocelis cruenta* [45]. Only the gametophytic phase was studied. The studied population presents a predominance of nonfructified plants in winter and a predominance of female gametophytes in spring/summer (Figure 4).

**Figure 5:**
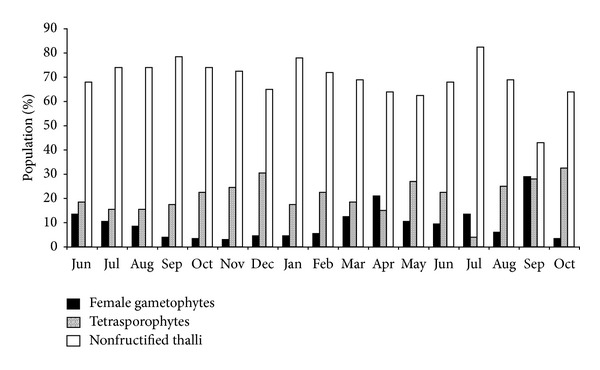
Population structure of *Chondracanthus teedei* var. *lusitanicus* in Buarcos bay.

*C. teedei *var.* lusitanicus* presents an isomorphic triphasic lifecycle [31, 46]. In Buarcos bay population, the nonfructified thalli were dominant in all samples (see Figure 5); the percentage varied from 43% (early autumn) to 82.5% (early summer). The female gametophytes were present in all samples, varying the proportion of 3% (late autumn) to 29% (late summer). The tetrasporophytes were also present in all samples, with a maximum of 32.5% in autumn and a minimum of 4% in summer. As compared to fructified thalli, namely, the female gametophytes bearing cystocarps (9.6 ± 1.7%, *n* = 17), the tetrasporophytes are, generally, more abundant (21 ± 1.7%, *n* = 17). The average percentage of nonfructified thalli was 69.4 ± 2.2% (*n* = 17). The data on seasonal variation in the percentage of individuals of each generation have statistical significance (one-way ANOVA, *P* < 0.001).

**Figure 6:**
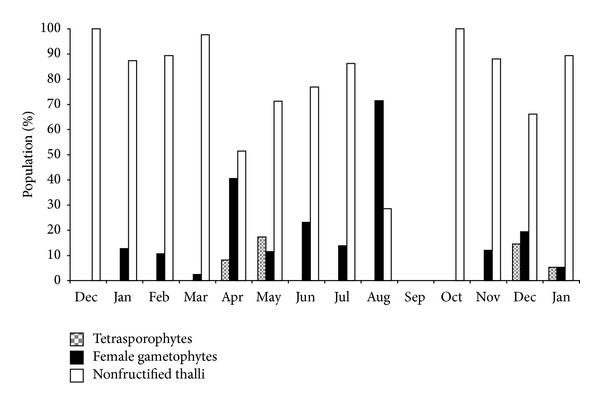
Population structure of *Calliblepharis jubata* in Buarcos bay.

*C. jubata* shows an isomorphic triphasic lifecycle [47], and the nonfructified plants were dominant in all samples, except in a summer sample (August); the percentage varied from 28.6% (summer) to 100.0% (autumn and early winter) (Figure 6). The female gametophytes were present in 11 of 13 samples, varying the proportion of 2.4% (late winter) to 71.4% (summer). The tetrasporophytes were present in 4 of 13 samples, with a maximum of 17.3% in spring and a minimum of 5.3% in winter. Comparing the fructified plants, particularly female gametophytes (20.3 ± 5.3%, *n* = 13), tetrasporophytes are always less abundant (11.3 ± 1.5%, *n* = 13). The average percentage of nonfructified thalli was 20.8 ± 5.6% (*n* = 13). The data on seasonal variation in the percentage of individuals of each generation have statistical significance (one-way ANOVA, *P* < 0.001).

The female gametophytes of *G. crenulatus* (species with a digenetic lifecycle) present reproductive structures known as tetrasporoblasts [46, 48, 49]; these structures appear as external wart-like excrescences. All the collected thalli showed tetrasporoblasts. *A. devoniensis* presents a heteromorphic triphasic lifecycle with crustose tetrasporophytes [46, 50]. Only the gametophytic phase was studied. The cystocarps were present in the gametophytes sampled in December, January, and March.

Our results are similar to those presented in other works on the North Atlantic [17, 37, 38, 49, 51] and the Iberian Peninsula carrageenophytes [1, 21].

### 3.4. Variation in Dry Weight and Carrageenan Content

The dry weight and carrageenan content average values are shown in Figure 1.The maximum carrageenan content in *C. crispus* (see Table 1) was found in a tretrasporic thalli sample in summer, with 53.2% of dry weight; a sample of female gametophytes presented the minimum value in late autumn, with 10% of dry weight. The data on seasonal variation of carrageenan content show statistical significance (one-way ANOVA, *P* < 0.01).

The maximum carrageenan content in *G. pistillata* (Table 1) was found in a sample of female gametophytes, with 59.7% of dry weight in late spring; a sample of heterosporic thalli presented the minimum value in late autumn, with 22.7% of dry weight. The data on seasonal variation of carrageenan content show statistical significance (one-way ANOVA, *P* < 0.001).

The population of *M. stellatus* is composed of about 50% of female gametophytes and 50% of nonfructified thalli, both showing a similar maximum carrageenan content (see Table 1) in summer, with about 41% dry weight. However, the average carrageenan content (20.7 ± 2.0%, *n* = 14) is relatively low, when compared to other carrageenophytes from Buarcos bay (Figure 1). The data on seasonal variation of carrageenan content show statistical significance (one-way ANOVA, *P* < 0.001).

Dry matter in *C. teedei *var.* lusitanicus* (Figure 1) varied between 12.1 ± 1.5% (*n* = 3) in late autumn and 17.5 ± 0.8% (*n* = 3) in winter. The maximum carrageenan content (58%) was found in a tetrasporophyte sample collected in summer (Table 1) and the minimum content was found in a nonfructified sample, with 23% in winter. The overall phycocolloid content was minimum (26.4 ± 2.5%, *n* = 3) in winter and maximum (43.6 ± 12.5%, *n* = 3) in early summer. The data on seasonal variation of dry weight and carrageenan content have statistical significance (one-way ANOVA, *P* < 0.001 and *P* < 0.05, resp.).

The average dry weight in *C. acicularis* ranged from 18.3 ± 0.2% (*n* = 3) in late spring to 33.0 ± 2.7% (*n* = 3) in autumn. The average dry weight was 24.8 ± 2.7 (*n* = 13). The maximum carrageenan content (61.1%) was found in a nonfructified thalli sample collected in summer (Table 1), and the minimum content was found in a female gametophyte sample, with 21.7% in autumn. The overall phycocolloid content was minimum (29.4 ± 4.3%, *n* = 3) in autumn and maximum (55.5 ± 4.6%, *n* = 3) in summer. The data on seasonal variation of dry weight and carrageenan content have statistical significance (one-way ANOVA, *P* < 0.001).

All the three remaining species, *C. jubata*, *G. crenulatus,* and *A. devoniensis*, are producers of iota-carrageenan and have been studied in other countries [49, 51, 52]. In general, our results are in accordance with those studies. The carrageenan content of these species is relatively low, varied from 10.1% in *A. devoniensis* to 13% in *C. jubata*, passing by 12.4% in *G. crenulatus*. Furthermore, the cover (Figure 2) and biomass are very low.

### 3.5. Carrageenan Composition

The ground seaweed samples FTIR-ATR spectra (not shown) of *C. crispus*, *M. stellatus*, and *G. pistillata* (female gametophytes) and the nonfructified plants of *C. crispus* exhibit strong absorption bands in the region of 930 cm^−1^ (DA) and the region of 845 cm^−1^ (G4S), typical of the kappa-carrageenan. These spectra have low absorbance in the region 805 cm^−1^ (DA2S), which means the presence of iota-carrageenan [8]. Female gametophytes of *C. crispus*, *M. stellatus*, and *G. pistillata* and the nonfructified plants of *C. crispus* ground seaweed FT-Raman spectra show two bands in the region 807 cm^−1^ (DA2s) and 850 cm^−1^ (G4S), typical of kappa*/*iota-hybrid carrageenans. The occurrence of additional peaks 821 cm^−1^ (G/D6S), 830 cm^−1^ (G/D2S), and 870 cm^−1^ (G/D6S) shows the presence of mu- and nu-carrageenan and biological precursors of kappa- and iota-carrageenan, respectively [8]. Our results agree with those obtained in other studies conducted with *C. crispus* [7, 53–55],* M. stellatus* [7, 56, 57], and *G. pistillata* [58–60].

In female gametophytes and nonfructified thalli of *C. teedei* var. *lusitanicus,* the FTIR-ATR spectra show strong absorption at 930 (DA) and 845 cm^−1^ (G4S) and median absorption in the band 805 cm^−1^ (DA2S). Additional peaks at 867 cm^−1^ (G/D6S), 825 cm^−1^ (G/D2S), and 820 cm^−1^ (G/D6S), with little intensity, correspond to the presence of carrageenan precursors (mu and nu). The presence of bands at 820 cm^−1^, 825 cm^−1^, and 867 cm^−1^, corresponding to the existence of precursors, is more evident in the FT-Raman spectra. These results agree with those obtained in other studies conducted with *C. teedei* [7, 8, 31, 61].

For the species *A. devoniensis*, *G. crenulatus,* and *C. jubata*, the FTIR-ATR spectra show absorption bands at 930, 845, and 805 cm^−1^, which represent the characteristic triplet of the fraction iota, when dominant in a hybrid carrageenan. The FT-Raman spectra of *A. devoniensis* and *G. crenulatus* show two bands in the region 807 cm^−1^ (DA2S) and 850 cm^−1^ (G4S), typical of hybrid kappa/iota carrageenans. The peaks related to the carrageenan precursors, mu and nu, are 821 cm^−1^ (G/D6S), 830 cm^−1^ (G/D2S), and 870 cm^−1^ (G/D6S) [8].

The intensity of the resonances in ^1^H-NMR spectra [14, 34] was used in this work in order to quantify the different carrageenan fractions (see Table 1). The alkali-extracted carrageenans showed lower sulphate content and a decrease in galactose to the benefit of 3,6-anhydrogalactose. This corresponds to the conversion of the 4-linked galactose-6-sulfate in native samples to anhydrogalactose in the alkali-extracted carrageenans. Thus, the carrageenan precursor's mu and nu were converted into kappa- and iota-carrageenan, respectively [31].

## 4. Conclusion

Several investigators [49, 51, 52, 54, 58] have described seasonal fluctuations of carrageenan quantity and compositions in some Gigartinales. In this study, most of the seaweeds present high carrageenan contents in summer (*C. crispus*, *C. teedei* var. *lusitanicus*, *G. pistillata*, *C. jubata,* and *G. crenulatus*); however, the maximum carrageenan content in *C. acicularis* and *A. devoniensis* is found in autumn/winter. 

In *C. crispus,* the carrageenan content was low in autumn and winter, a small increase occurred in early spring (April), and the largest carrageenan content was recorded in samples collected in summer (July). In *G. pistillata,* the carrageenan content was low in autumn and winter, a large increase occurred in early spring (March), and the biggest carrageenan content was recorded in samples collected in spring (June) and summer (July and August). In *C. acicularis,* carrageenan content was low in autumn and winter, a small increase occurred in early spring (March), and the highest carrageenan content was recorded in samples collected in summer (July). In *M. stellatus,* the carrageenan content was low in winter and spring, a small increase occurred in early summer (June), and the highest carrageenan content was recorded in samples collected at the end of summer (September). In *A. devoniensis,* the carrageenan content was low throughout the study period and a small increase occurred only in summer (June). In *G. crenulatus,* the carrageenan content was low during the autumn, and a small increase occurred in winter; the spring samples and in particular those of summer have a higher carrageenan content. Finally, in *C. jubata* the yield was low in autumn and winter; the highest carrageenan content was recorded in samples collected in spring (May).

So, by the combination of high biomass and carrageenan content available in summer, we can conclude that this is the best period to harvest the Portuguese dominant carrageenophytes, with the exception of *C. acicularis*, that will have to be harvested in autumn/winter. Other studies carried out in North Atlantic coasts showed an increase in carrageenan content during summer and a decrease in winter, namely, in *C. crispus* [54], *C. jubata* [51, 52], and *G. crenulatus* [49]. 

In relation to the nature of the phycocolloid, our vibrational and resonance spectroscopic analysis showed that the Portuguese carrageenophytes studied seem to present a similar composition to that found in other species of Cystocloniaceae, Gigartinaceae, and Phyllophoraceae families [5].

In conclusion, some species found in the central north coast of Iberian Peninsula could be used for industrial applications. Kappa, kappa-iota hybrid, and lambda fractions can be provided by harvesting *C. crispus*, *M. stellatus*, *C. teedei* var. *lusitanicus,* and *C. acicularis*. However, responsible harvesting of natural populations must be always the norm, because the nonsustainable procedures can have severe economic and environmental impacts. On the other hand and due to its limited stock in the western coast, *G. pistillata *(source of hybrid kappa-iota and xi-lambda carrageenans), *C. jubata*, *G. crenulatus,* and *A. devoniensis* (sources of iota-carrageenan) should be, in future, objects of culture essays in order to research their potentialities in biomass production.

## Figures and Tables

**Table 1:** Biomass, yield, and carrageenan composition.

Family	Species	Biomass (gm^−2^) (dry weight)	Lifecycle phase	Harvest season	Carrageenan			
					Yield^(1)^	Letter code^(2)^	Alkali-extracted^(3)^ (%mol)	Native^(4)^
Cystocloniaceae	*Calliblepharis jubata *	10.3 ± 1.3 (*n* = 15)	NF	Spring	20.0 ± 1.7 (*n* = 3)	G4S-DA2S	100 *ι*	*ι* (*ν*)
	*C. jubata *	11.0 ± 0.5 (*n* = 15)	T	Spring	28.4 ± 1.3 (*n* = 3)	G4S-DA2S, G4S-DA	98 *ι*, 2 *κ*	*ι* − *κ* (*ν*)
	*C. jubata *	11.3 ± 0.3 (*n* = 15)	FG	Spring	40.4 ± 3.2 (*n* = 3)	G4S-DA2S	100 *ι*	*ι* (*ν*)

Gigartinaceae	*Chondracanthus acicularis *	24.2 ± 1.8 (*n* = 15)	NF	Summer	61.1 ± 2.9 (*n* = 3)	G4S-DA, G4S-DA2S	—	*κ* − *ι* (*μ*/*ν*)
	*C. acicularis *	37.5 ± 2.6 (*n* = 3)	T	Summer	36.6 ± 3.7 (*n* = 3)	G2S-D2S, G2S-DA2S	59 *ξ*, 41 *θ*	*ξ* − *θ*
	*C. acicularis *	24.3 ± 1.6 (*n* = 15)	FG	Late summer	49.8 ± 2.4 (*n* = 3)	G4S-DA, G4S-DA2S	61 *κ*, 34 *ι*, 5 pyruvate	*κ* − *ι* (*μ*/*ν*)
	*Chondracanthus teedei* var. *lusitanicus *	12.2 ± 2.2 (*n* = 15)	NF	Late summer	41.0 ± 4.4 (*n* = 3)	G4S-DA, G4S-DA2S	56 *κ*, 44 *ι*	*κ* − *ι* (*μ*/*ν*)
	*C. teedei* var. *lusitanicus *	15.3 ± 1.7 (*n* = 15)	T	Summer	58.0 ± 8.0 (*n* = 3)	G2S-D2S, G2S-DA2S	67 *ξ*, 33 *θ*	*ξ* − *θ*
	*C. teedei *var. *lusitanicus *	14.1 ± 1.7 (*n* = 15)	FG	Late summer	57.4 ± 6.0 (*n* = 3)	G4S-DA, G4S-DA2S	58 *κ*, 42 *ι*	*κ* − *ι* (*μ*/*ν*)
	*Chondrus crispus *	22.0 ± 0.7 (*n* = 15)	NF	Late summer	36.0 ± 1.8 (*n* = 3)	G4S-DA, G4S-DA2S	64 *κ*, 36 *ι*	*κ* − *ι* (*μ*)
	*C. crispus *	23.3 ± 0.6 (*n* = 15)	FG	Summer	36.8 ± 2.2 (*n* = 3)	G4S-DA, G4S-DA2S	70 *κ*, 30 *ι*	*κ* − *ι* (*μ*)
	*C. crispus *	24.2 ± 0.5 (*n* = 15)	T	Summer	58.0 ± 2.8 (*n* = 3)	G2S-D2S, 6S	100 *λ*	*λ*
	*Gigartina pistillata *	22.9 ± 0.7 (*n* = 15)	T	Late summer	57.0 ± 2.8 (*n* = 3)	G2S-D2S, G2S-D2S, 6S	—	*ξ* − *λ*
	*G. pistillata *	21.2 ± 0.8 (*n* = 15)	H (*⊕*)	Summer	58.5 ± 2.4 (*n* = 3)	G2S-D2S, G2S-D2S, 6S	—	*ξ* − *λ*
	*G. pistillata *	20.4 ± 0.5 (*n* = 15)	FG	Summer	59.7 ± 2.7 (*n* = 3)	G4S-DA, G4S-DA2S	48 *κ*, 45 *ι*, 7 pyruvate	*κ* − *ι* (*μ*/*ν*)
	*G. pistillata *	20.8 ± 0.6 (*n* = 15)	H (♀)	Summer	58.5 ± 2.6 (*n* = 3)	G4S-DA, G4S-DA2S	49 *κ*, 45 *ι*, 6 pyruvate	*κ* − *ι* (*μ*/*ν*)

Phyllophoraceae	*Ahnfeltiopsis devoniensis *	22.8 ± 1.2 (*n* = 15)	G	Summer	13.6 ± 0.5 (*n* = 3)	G4S-DA2S, G4S-DA	82 *ι*, 18 *κ*	*ι* − *κ* (*ν*)
	*Gymnogongrus crenulatus *	18.4 ± 1.3 (*n* = 15)	TB	Late spring	23.3 ± 1.4 (*n* = 3)	G4S-DA, G4S-DA2S	64 *κ*, 29 *ι*, 7 pyruvate	*κ* − *ι* (*μ*/*ν*)
	*Mastocarpus stellatus *	28.4 ± 0.6 (*n* = 15)	NF	Late summer	41.4 ± 2.0 (*n* = 3)	G4S-DA, G4S-DA2S	62 *κ*, 36 *ι*, 2 pyruvate	*κ* − *ι* (*μ*/*ν*)
	*M. stellatus *	20.9 ± 2.1 (*n* = 15)	FG	Late summer	41.0 ± 2.0 (*n* = 3)	G4S-DA, G4S-DA2S	59 *κ*, 41 *ι*	*κ* − *ι* (*μ*/*ν*)

T: tetrasporophyte; FG: female gametophyte; G: gametophyte; NF: nonfructified thalli; TB: tetrasporopblastic thalli; H: heterosporic thalli: (♀ cistocarpic branch; *⊕* tetrasporic branch); ^(1)^ yields (maximum value found) expressed as a percentage of dry weight ± standard error; ^(3)^composition determined by ^1^H-NMR; ^(4)^composition determined by FTIR-ATR and FT-Raman analysis of ground seaweed samples; the carrageenans are identified according to the Greek lettering system and letter code ^(2)^proposed by Knutsen et al. [3]; the letters between parentheses ( ) correspond to the biological precursors of the carrageenans, present in native carrageenan samples (or ground seaweed). Kappa (*κ*), Mu (*μ*), Iota (*ι*), Nu (*ν*), Theta (*θ*), Lambda (*λ*), and Xi (*ξ*).

## References

[1] Pereira L, van de Velde F (2011). Portuguese carrageenophytes: carrageenan composition and geographic distribution of eight species (Gigartinales, Rhodophyta). *Carbohydrate Polymers*.

[2] van de Velde F, de Ruiter GA, Steinbüchel A, de Baets S, VanDamme EJ (2002). Carrageenan. *Biopolymers*.

[3] Knutsen SH, Myslabodski DE, Larsen B, Usov AI (1994). A modified system of nomenclature for red algal galactans. *Botanica Marina*.

[4] Craigie JS, Cole KM, Sheath RG (1990). Cell walls. *Biology of the Red Algae*.

[5] Chopin T, Kerin BF, Mazerolle R (1999). Phycocolloid chemistry as a taxonomic indicator of phylogeny in the Gigartinales, Rhodophyceae: a review and current developments using Fourier transform infrared diffuse reflectance spectroscopy. *Phycological Research*.

[6] Rudolph B, Martin RE (2000). Seaweed products: red algae of economic significance. *MarIne & Freshwater Products Handbook*.

[7] Pereira L, Mesquita JF (2003). Carrageenophytes of occidental Portuguese coast: 1-spectroscopic analysis in eight carrageenophytes from Buarcos bay. *Biomolecular Engineering*.

[8] Pereira L, Amado AM, Critchley AT, van de Velde F, Ribeiro-Claro PJA (2009). Identification of selected seaweed polysaccharides (phycocolloids) by vibrational spectroscopy (FTIR-ATR and FT-Raman). *Food Hydrocolloids*.

[9] Pereira L, Critchley AT, Amado AM, Ribeiro-Claro PJA (2009). A comparative analysis of phycocolloids produced by underutilized *versus* industrially utilized carrageenophytes (Gigartinales, Rhodophyta). *Journal of Applied Phycology*.

[10] Bixler HJ, Johndro K, Falshaw R (2001). Structure and performance of commercial extracts II. Performance in two simulated dairy applications. *Food Hydrocolloids*.

[11] Wong KF, Craigie JS (1978). Sulfohydrolase activity and carrageenan biosynthesis in *Chondrus crispus* (Rhodophyceae). *Plant Physiology*.

[12] de Ruiter GA, Richard O, Rudolph B Sulfohydrolases, corresponding amino acid and nucleotide sequences, sulfohydrolase preparations, processes, and products thereof.

[13] Aguilan JT, Broom JE, Hemmingson JA (2003). Structural analysis of carrageenan from farmed varieties of Philippine seaweed. *Botanica Marina*.

[14] van de Velde F, Knutsen SH, Usov AI, Rollema HS, Cerezo AS (2002). ^1^H and ^13^C high resolution NMR spectroscopy of carrageenans: application in research and industry. *Trends in Food Science and Technology*.

[15] Pereira L, Pomin VH (2011). A review of the nutrient composition of selected edible seaweeds. *Seaweed: Ecology, Nutrient Composition and MedicInal Uses*.

[16] Briand X, Guiry MD, Blunden G (1991). Seaweed harvesting in Europe. *Seaweed, Resources In Europe—Uses and Potential*.

[17] Perez R, Kaas R, Campello F, Arbault S, Barbaroux O (1992). *La Culture des Algues Marines Dans le Monde*.

[18] Palminha FP (1971). Exploração e utilização de algas marinhas na plataforma portuguesa e nas ilhas do Arquipélago dos Açores. *Junta Nacional de Fomento das Pescas*.

[19] Sousa-Pinto I, Araújo R, Critchley A, Ohno M, Largo D (2006). The seaweed resources of Portugal. *World Seaweed Resources: An Authoritative Reference System*.

[20] Santos R, Duarte P (1991). Marine plant harvest in Portugal. *Journal of Applied Phycology*.

[21] García Tasende M, Cid M, Fraga MI (2012). Spatial and temporal variations of *Chondrus crispus* (Gigartinaceae, Rhodophyta) carrageenan content in natural populations from Galicia (NW Spain). *Journal of Applied Phycology*.

[22] Tasende MG, Cid M, Fraga MI (2013). Qualitative and quantitative analysis of carrageenan content in gametophytes of *Mastocarpus stellatus* (Stackhouse) Guiry along Galician coast (NW Spain). *Journal of Applied Phycology*.

[23] McHugh DJ (2003). A guide to the seaweed industry.

[24] Bixler HJ, Porse H (2011). A decade of change in the seaweed hydrocolloids industry. *Journal of Applied Phycology*.

[25] Pereira L, Critchley AT, Ohno M, Largo DB (2006). Identification of phycocolloids by vibrational spectroscopy. *World Seaweed Resources—An Authoritative Reference System*.

[26] Pereira L, Gheda FS, Ribeiro-Claro PJA (2013). Analysis by vibrational spectroscopy of seaweed polysaccharides with potential use in food, pharmaceutical and cosmetic industries. *International Journal of Carbohydrate Chemistry*.

[27] Krause-Jensen D, Carstensen J, Dahl K (2007). Total and opportunistic algal cover in relation to environmental variables. *Marine Pollution Bulletin*.

[28] Neto JM, Gaspar R, Pereira L, Marques JC (2012). Marine Macroalgae Assessment Tool (MarMAT) for intertidal rocky shores. Quality assessment under the scope of the European Water Framework Directive. *Ecological Indicators*.

[29] Braga MRA (1990). Reproductive characteristics of *Gigartina teedii* (Roth) Lamouroux (Rhodophyta, Gigartinales), a turf-forming species—field and laboratory culture studies. *Botanica Marina*.

[30] Chopin T Marine Biodiversity Monitoring: protocol for monitoring of seaweeds. http://macoi.ci.uc.pt/include/downloadContentDoc.php?id=12.

[31] Pereira L, Mesquita JF (2004). Population studies and carrageenan properties of *Chondracanthus teedei* var. *lusitanicus* (Gigartinaceae, Rhodophyta). *Journal of Applied Phycology*.

[32] Pereira L, Sousa A, Coelho H, Amado AM, Ribeiro-Claro PJA (2003). Use of FTIR, FT-Raman and 13C-NMR spectroscopy for identification of some seaweed phycocolloids. *Biomolecular Engineering*.

[33] Zar JH (1996). *Biostatistical Analysis*.

[34] van de Velde F, Pereira L, Rollema HS (2004). The revised NMR chemical shift data of carrageenans. *Carbohydrate Research*.

[35] Chen LCM, McLachlan J (1971). The life history of *Chondrus crispus* in culture. *Canadian Journal of Botany*.

[36] Guiry MD (1981). *Chondrus crispus* Stackhouse “T4” is a male clone (Rhodophyta). *Phycologia*.

[37] Brown MT, Neish A, Harwood D (2004). Comparison of three techniques for identifying isomorphic phases of *Chondrus crispus* (Gigartinaceae). *Journal of Applied Phycology*.

[38] Scrosati R, Mudge B (2004). Persistence of gametophyte predominance in *Chondrus crispus* (Rhodophyta, Gigartinaceae) from Nova Scotia after 12 years. *Hydrobiologia*.

[39] Scrosati R, Garbary DJ, McLachlan J (1994). Reproductive ecology of *Chondrus crispus* (Rhodophyta, Gigartinales) from Nova Scotia, Canada. *Botanica Marina*.

[40] Kim DH (1976). A study of the development of cystocarps and tetrasporangial sori in Gigartinaceae (Rhodophyceae, Gigartinales). *Nova Hedwigia*.

[41] Hommersand MH, Guiry MD, Fredericq S, Leister GL (1993). New perspectives in the taxonomy of the Gigartinaceae (Gigartinales, Rhodophyta). *Hydrobiologia*.

[42] Hommersand M, Fredericq S, Cabioch J (1992). Developmental morphology of *Gigartina pistillata* (Gigartinaceae, Rhodophyta). *Phycologia*.

[43] Isaac WE, Simons SM (1954). Some observations on *Gigartina pistillata* (Gmel.) Stackh. from Port Alfred with a record of plants bearing both tetraspores and carpospores. *Journal of South African Botany*.

[44] Pereira L, Mesquita JF, Dias JDS Optical and electron microscope study of heterosporic thalli (carpospores/tetraspores) in *Gigartina pistillata* (Gmel.) Stackh. (Rhodophyta).

[45] Guiry MD, West JA, Kim DH, Masuda M (1984). Reinstatement of the Genus *Mastocarpus* Kutzing (Rhodophyta). *Taxon*.

[46] Pereira L, Heimann K, Katsaros C (2012). Cytological and cytochemical aspects in selected carrageenophytes (Gigartinales, Rhodophyta). *Advances in Algal Cell Biology*.

[47] Dixon PS, Irvine LM (1995). *Seaweeds of the British Isles, Vol. I—Rhodophyta, Part 1—Introduction, Nemaliales, Gigartinales*.

[48] Gayral P (1966). Les Algues de côtes françaises (manche et atlantique), notions fondamentales sur l'écologie, la biologie et la systématique des algues marines.

[49] Mathieson AC, Emerich Penniman C, Tveter-Gallagher E (1984). Phycocolloid ecology of underutilized economic red algae. *Hydrobiologia*.

[50] Maggs CA, Douglas SE, Fenety J, Bird CJ (1992). A molecular and morphological analysis of the *Gymnogongrus devoniensis* (Rhodophyta) complex in the North Atlantic. *Journal of Phycology*.

[51] Cosson J, Deslandes E, Braud JP (1990). Preliminary approach to the characterization and seasonal variation of carrageenans from four Rhodophyceae on the Normandy coast (France). *Hydrobiologia*.

[52] Zinoun M, Cosson J (1996). Seasonal variation in growth and carrageenan content of *Calliblepharis jubata* (Rhodophyceae, Gigartinales) from the Normandy coast, France. *Journal of Applied Phycology*.

[53] Kopp J (1978). *Contribuition à l'étude de l'algue rouge Chondrus crispus Stackh. Biochimie des carraghénanes [Ph.D. thesis]*.

[54] Kopp J, Perez J (1979). Contribuition à l'étude de l'álgue rouge Chondrus crispus Stackh. Relation entre la croissance, la potentialité sexuelle, la quantité e la composition de carraghénanes. *Revue des Travaux de l'Institut des Peches Maritimes*.

[55] Chopin T, Floch J-Y (1992). Eco-physiological and biochemical study of two of the most contrasting forms of *Chondrus crispus* (Rhodophyta, Gigartinales). *Marine Ecology Progress Series*.

[56] Hilliou L, Larotonda FDS, Sereno AM, Gonçalves MP (2006). Thermal and viscoelastic properties of *κ*/*ι*-hybrid carrageenan gels obtained from the Portuguese seaweed *Mastocarpus stellatus*. *Journal of Agricultural and Food Chemistry*.

[57] Hilliou L, Larotonda FDS, Abreu P, Ramos AM, Sereno AM, Gonçalves MP (2006). Effect of extraction parameters on the chemical structure and gel properties of *κ*/*ι*-hybrid carrageenans obtained from *Mastocarpus stellatus*. *Biomolecular Engineering*.

[58] Amimi A, Mouradi A, Bennasser L, Givernaud T (2007). Seasonal variations in thalli and carrageenan composition of *Gigartina pistillata* (Gmelin) Stackhouse (Rhodophyta, Gigartinales) harvested along the Atlantic coast of Morocco. *Phycological Research*.

[59] Amimi A, Mouradi A, Givernaud T, Chiadmi N, Lahaye M (2001). Structural analysis of *Gigartina pistillata* carrageenans (Gigartinaceae, Rhodophyta). *Carbohydrate Research*.

[60] Pereira L, Mesquita JF, Long CA, Anninos P Cytochemical studies on Portuguese carrageenophytes (Gigartinales, Rhodophyta).

[61] Zinoun M, Cosson J, Deslandes E (1993). Influence of culture conditions on growth and physicochemical properties of carrageenans in *Gigartina teedii* (Rhodophyceae, Gigartinales). *Botanica Marina*.

